# Use of the Principles of Design Thinking to Address Limitations of Digital Mental Health Interventions for Youth: Viewpoint

**DOI:** 10.2196/11528

**Published:** 2019-01-14

**Authors:** Hanneke Scholten, Isabela Granic

**Affiliations:** 1 Behavioural Science Institute Radboud University Nijmegen Netherlands

**Keywords:** anxiety, depression, design thinking, e-mental health, youth

## Abstract

Numerous reviews and meta-analyses have indicated the enormous potential of technology to improve the appeal, effectiveness, cost, and reach of mental health interventions. However, the promise of digital mental health interventions for youth has not yet been realized. Significant challenges have been repeatedly identified, including engagement, fidelity, and the lack of personalization. We introduce the main tenets of design thinking and explain how they can specifically address these challenges, with an entirely new toolbox of mindsets and practices. In addition, we provide examples of a new wave of digital interventions to demonstrate the applicability of design thinking to a wide range of intervention goals. In the future, it will be critical for scientists and clinicians to implement their scientific standards, methods, and review outlets to evaluate the contribution of design thinking to the next iteration of digital mental health interventions for youth.

## Background

The prevalence of mental health problems has significantly increased among children (aged 5-10 years) and adolescents (aged 10-24 years [1]) [2-5], and the current prevalence rate of mental disorders is estimated to be 13.4% [2-5]. According to the latest update from the World Health Organization [6], half of all mental health disorders in adulthood start by the age of 14 years and three-quarters, by the mid-20s. As a result of the increasing overall prevalence rates, increases in the rates of mental health concerns for young people specifically [1], and a constant number of available treatments over time, 64%-87% of mental health issues in young people are undetected and untreated [6,7-9].

The rapid growth of technological innovations has been welcomed as an unprecedented opportunity to address the increasing gap between demand and supply of mental health services by many in the mental health research and practice communities [10,11]. Several reviews have indicated the enormous potential of technology to improve the effectiveness, efficiency, cost, reach, personalization, and appeal of mental health interventions [12-16]. Under the rubric of “e-mental health,” such advantages are proposed to rely on the ubiquitous role of interactive media in the daily lives of young people [17]. However, the effectiveness of digital technology in reducing the burden of mental health at a population level is progressing slowly at best [18-21]. It remains uncertain whether the hype and promise of e-mental health solutions will actually be realized [15,22].

In the current paper, we summarize and critique the available evidence on the efficacy of digital interventions for young people, with a focus on those targeting anxiety and depression (treatment and indicated prevention). There are many excellent reviews and meta-analyses summarizing the efficacy of digital interventions in the literature; this viewpoint does not attempt to do the same. Instead, we briefly summarize the evidence indicating poor outcomes, especially for youth. We have only included effects based on postintervention measurements, because most meta-analyses did not have the power to reliably conclude the effects on follow-up measurements. Thereafter, we outline an altogether new approach that has the potential to dramatically improve digital tools for youth mental health by using principles from the discipline of design. Design thinking (DT) is usually considered outside the purview of scientific research; however, we argue that this cross-disciplinary approach may be key to galvanizing progress. Three tenets of DT are introduced here, and preliminary empirical evidence from our own laboratory demonstrates both the opportunities and challenges of this new approach. Finally, as this design framework is new in the mental health arena, we end this paper with recommendations for systematic programs of research that directly test its impact on effectiveness and its implications for implementation.

## Outcome Research on Digital Mental Health Interventions

The widespread availability of digital technology has led to a proliferation of digital mental health (DMH) interventions. A substantial part of DMH approaches target depression and anxiety and are based on cognitive behavioral therapy (CBT), originally developed several decades ago for face-to-face treatment and adjusted for self-help books and manuals [12]; we focus our review and recommendations on this class of DMH interventions. There is also a growing body of promising research on virtual reality interventions, especially in the clinical context [23], but it is not directly relevant to our current purposes.

Overall, the efficacy of DMH programs for depressed and anxious adults has been established by over 100 studies [12]. Based on the latest meta-analysis for anxiety disorders among adults [16], guided DMH interventions are more effective than waiting list, attention, information, or online discussion groups. For depression [24], DMH interventions were favored over waiting lists only. There are limited data available to compare DMH interventions and active treatment or placebo control groups; however, the available data suggest that DMH interventions are more effective than other interventions (with smaller effect sizes than those of a waiting list). Other studies also suggest that guided DMH interventions do as well as active treatment control groups (ie, face-to-face CBT) for anxiety [16] and depression [13].

There is still considerable variability in the outcomes, and the inclusion (or exclusion) of human guidance could be one of the key factors that explain this variance. The influence of human guidance on DMH interventions for adult anxiety and depression is still debated [25,26]. Some meta-analyses report equal effects of guided and unguided DMH interventions for anxiety [16] (but with very low-quality evidence) and depression [24], whereas other studies have shown that guided DMH interventions outperform unguided interventions [27-29]. Based on a recent meta-analysis on depression symptoms [30], unguided DMH interventions are more effective than waiting list, attention placebo, no treatment, or treatment as usual; however, its effect is much smaller than that of guided DMH interventions. Importantly, Ebert and Baumeister [25] argue that these meta-analyses are solely based on randomized controlled trials (RCTs), which require a high level of commitment and adherence from patients, unlike the conditions in routine clinical care. It is likely that the reported effect sizes of unguided DMH interventions under laboratory settings are overestimated for their potential in routine clinical care. Thus, despite optimism about the potential of DMH tools as standalone interventions for adults, human involvement in its delivery and monitoring seems to be an important mediator of success for routine clinical care outside the laboratory.

The results are less optimistic for the comparably smaller body of evidence available for children and adolescents. Based on the most recent meta-review by Hollis and colleagues [15], overall effect sizes for youth are moderate to large for anxiety disorders and small to moderate for depression disorders when DMH approaches are compared to a waitlist group. However, analyses comparing DMH interventions for both anxiety and depression with active nontherapeutic controls have generally failed to show superiority of DMH interventions [31,32]. Noninferiority trials comparing DMH interventions to face-to-face CBT in order to determine whether a new intervention is therapeutically similar to an existing effective treatment [33] showed that most DMH approaches were as effective as face-to-face CBT [31,34-36]. In contrast, Pennant and colleagues [32] showed that face-to-face CBT was more effective than DMH interventions. Thus, although the available evidence is not yet conclusive for youth-focused studies, some human guidance seems to be important for the effects on anxiety and depression.

The role of human guidance in youth-focused studies is difficult to ascertain, as the level of human support is poorly specified across trials [15]. However, one of the most recent studies [37] reported that the interventions in their meta-analysis that favored the DMH intervention group included face-to-face guidance, monitoring of engagement, or follow-up telephone calls by teachers and health professionals. Importantly, the quality of the youth-focused studies for DMH programs is generally low to moderate, which is most often a result of methodological flaws such as intervention heterogeneity in terms of content, dose, settings, or quality [15,38]. Furthermore, problems such as insufficient search processes of the literature, small sample sizes, differences at baseline in study samples, and publication bias play important roles in the quality of these studies [32,39]. In summary, there is reasonable support for the role of DMH tools in improving anxiety and depression problems in adults, but there are fewer promising results for children and adolescents. In youth-focused research, poor overall outcomes, heterogeneity of results, and poor quality of many studies prevent definitive conclusions.

## Limitations of DMH Interventions for Youth

Despite some promise, there is growing consensus about the limitations of DMH approaches, with almost every meta-analysis and systematic review (both with adult and youth samples) highlighting the same problems. First, high attrition rates and low adherence to protocols are consistently problematic, especially in unguided interventions [30,37,39,40] as compared to guided DMH interventions [37,41]. Considering only unguided digital interventions, a meta-analytic study by Karyotaki and colleagues [42] showed that almost 70% of participants dropped out before completing 75% of the intervention. Attrition and low adherence are even bigger challenges among children and adolescents; the younger the participant sample, the greater the dropout rate [37,42]. Välimäki and colleagues [37] showed that young people (between the ages of 10 and 24 years) in the digital intervention groups (most often, guided DMH interventions) left the study earlier than the control group participants. Thus, the use of guided digital interventions seems to be the best solution; however, the need for therapists compromises the often espoused advantage of DMH interventions—their scalability (ie, easily deployed across the globe to populations with different economic and ethnic backgrounds [15]).

Most importantly, DMH tools do not remotely approximate the level of attractiveness and interactivity to which young “digital natives” have grown accustomed [17,32,43]. In the first generation of DMH tools, most researchers and intervention scientists seem to have assumed that moving content online and providing youth the agency to navigate this content at their own pace and in their own context makes the content more engaging than that in conventional treatment approaches. However, in a vast majority of cases, the content of DMH interventions is not significantly changed from the manuals from which they were derived. In the understandable and commendable effort to remain “evidence-based,” most DMH interventions for youth are a little-more-than modified and uploaded CBT manuals and workbooks (eg, MoodGym, Cool Kids, Camp Cope-a-lot, and BRAVE; for a review discussing the evidence base of these DMH interventions, see [32,44]). It is likely that the digital incarnations of these CBT interventions are rendered even less engaging than their original format because they are less flexible and personalized. No therapist is available to maintain motivation for change, build trust and hope, and sensitively tailor the treatment to personal idiosyncrasies.

Many DMH interventions are based on a one-size-fits-all approach (eg, a linear progression with content released to all participants using time-based rules [44]), which has its advantages because it is systematic, but remains problematic because of its perceived inflexibility [45]. Young people, in particular, value self-reliance and control when accessing digital products [46] or mental health services [47], and current DMH interventions are often perceived as impersonal and unresponsive to their individual needs [15,43]. In addition, DMH interventions are content focused (ie, CBT techniques) and not user focused (ie, they are not designed around how and when young people prefer to engage with digital experiences) [48], resulting in a large disconnect between the world in which youth live and the content and style of DMH interventions. For the current and upcoming generation of youth who play video games and socialize online daily, the norm is digital experiences that are exquisitely designed to adjust to the pace, content preferences, and skill levels of their users [49]. Personalization is consistently mentioned as one of the biggest advantages of digital solutions, but personalization, dynamic adjustment, and tailoring have not been realized with DMH tools thus far [15,43,50].

The cognitive load of DMH programs seems to be an additional limitation especially for young people. Many e-mental health programs are overly pedantic, didactic, and cognitively focused [44], thereby potentially overloading children and youth who find this approach too difficult and inaccessible [51]. Homework assignments pose an additional problem, as they rely on the abilities of the child or adolescent to practice the CBT-based exercises and learn accordingly. Youth very often fail to adequately follow through on these offline homework assignments, because they simply do not understand them well enough to practice or are not motivated to do so [35,39]. The same practice and homework problems can arise in conventional CBT, of course, but in face-to-face treatments, therapists are present to motivate, encourage, answer questions, and keep clients accountable [12].

At this point, an important caveat is in order: We are by no means advocating exclusion of therapists, coaches, and teachers altogether, especially in serious, chronic mental health cases among youth. Our best outcomes for serious clinical youth cases may come from combining face-to-face interactions with digital intervention “homework,” in which young people practice the lessons they have learned in the comfort of their own home or on mobile devices embedded in their everyday lives (eg, “blended” approaches [52]). However, this digital homework still requires attention to be paid to the factors that motivate and engage users. For less severe mental health cases, DMH programs may serve as stand-alone interventions preventing at-risk youth from symptom aggravation.

In summary, a convincing set of reviews and meta-analyses suggest that the promises of digital solutions, especially those targeted at youth, have not yet been realized [11,12,37]. Specifically, the benefits of DMH interventions, including increased engagement and motivation, fidelity to intervention protocols, and opportunities for personalization [12,48,50,53], remain largely unrealized. Perhaps unsurprisingly, all the reviews we have summarized end with general recommendations for reflection and reform, urging future efforts to take engagement, retention, and fidelity more seriously. However, these critical reflections consistently stop at that point, providing no concrete, actionable solutions to address the limitations they revealed [54-57]. In the rest of this viewpoint, we elaborate on a set of guiding principles and concrete strategies to potentially address this impasse.

## Design Thinking: Novel Recommendations and Proposed Solutions

In the following section, we outline a design framework that has helped us reimagine the development of DMH solutions for children and adolescents. Our approach started by identifying the limitations of past DMH interventions for youth and attempting to directly address each of them. A major step toward such solutions derives from our work with applied video games for mental health. We previously provided a detailed empirical review [49] that supports the rationale for using digital games as intervention tools for young people. In short, well-designed applied games are intrinsically motivating, offer a strong sense of agency, and are simply fun. They also provide a compelling virtual playground to not only gain knowledge, but also *practice* skills. Finally, applied games can overcome the stigma associated with traditional and self-help interventions.

We are not the first to suggest that applied games are useful intervention approaches. A zeitgeist has emerged in the medical and educational fields for applied or “serious” games as tools for enhancing medical care [58-61]. Although much less work has been done with serious games for mental health as compared to other conditions, several game-based interventions have been developed [62-65]. A large part of our message is that not all digital interventions, including games, are designed equally, and most serious games have the same general limitations that we outlined for DMH interventions. Our solution has been to adopt a DT framework, which provides a general cohesive set of principles and recommendations for DMH delivery.

### Defining Design Thinking

Before defining DT, it is important to understand that this approach is not simply about making products or services more attractive, pretty, or graphically sophisticated. It often involves some degree of esthetic improvement, but fundamentally, DT is both a mindset and a set of practices that are solution based. The business community as well as the healthcare, transportation, and creative industries have benefited enormously from the adoption of DT [56,66-70]. Compared to scientific practices in which data are “objective,” observable facts that are tested against *a priori* hypotheses, DT is a fundamentally subjective practice that focuses on discovering the emotional needs of users, their idiosyncratic contexts, their motivational concerns, and other related entities. DT aims to build a practical product or service that serves a very specific need.

We do not suggest that DT is enough as a stand-alone practice to address the concerns we have listed about DMH interventions for youth. However, combined with rigorous scientific standards and methodologies, this cross-disciplinary approach holds a great deal of promise. There are three core tenets of DT [67,68]: Empathy, which is a human-centered approach that keeps the emotional, motivational, and functional needs of users at the center of the development process; Multidisciplinary Ideation, which involves solutions generated by cross-disciplinary teamwork and collaboration; and Experimentation, which is the practice of rapid prototyping and iteratively testing products or services with target users during, rather than after, the development phase. These terms have varied meanings in psychology, psychiatry, and clinical practice, but have very specific meanings in the discipline of design, as elaborated in the next section.

#### Empathy

At its core, empathy-based design is a human-centered approach that answers the question “who is it for?” rather than “what does the product look like and contain?” Empathy seems like a fuzzy, unscientific lens through which evidence-based practice is considered, but it is the most crucial and, perhaps, least understood or integrated practice in the development of digital interventions. Empathy-driven design seeks to optimize user engagement, immersion, and motivation and as such, it has the potential to address key limitations of conventional DMH approaches (ie, high attrition, low adherence, and cognitive load). Instead of starting with the common premise, “we’re going to design an app that does X,” empathic design begins with “we’re going to solve X for a specific population” and thus helps developers expand beyond the exclusive content focus of DMH programs (eg, CBT techniques) toward user concerns (eg, a young person’s preferences and digital habits).

Beyond understanding the demographics, personalities, and preferences of individual users, empathic design keeps the whole end-to-end user *experience* in mind. Applied to youth mental health, user experience can be conceptualized according to these questions: (1) How are young people going to find an intervention, game, or service? (2) When they find it, does that digital ecosystem motivate them to keep discovering more, or does it shut them down? (c) Are there positive expectations for change embedded in a growth mindset? [71,72] (3) How long after they purchase or freely download the product, service, or game will it provide feedback about progress, and how will that make users feel? (4) Can they share it with like-minded peers and concerned adults? (5) Will it be updated with new content to keep them interested over longer periods? (6) When the experience ends, is there a feeling of mastery?

An empathy-driven approach also includes *participatory design*: We not only design *for* young people but *with* them as well and do so from the start of the design process. As digital natives [73], young people are using interactive media and technology almost from birth [17] and on a daily basis. By the time they engage with any particular DMH product, they have grown accustomed to interacting with highly engaging, sophisticated, and immersive contexts. If digital interventions are to stand a chance of improving the mental health of youth in the coming decades, they will need to be designed to stimulate and retain users’ attention. The first step towards ensuring that this will happen is to invite these users to codevelop products aimed at their cohort. Several other researchers have suggested the importance of recruiting young people in the development process. This practice has been referred to as participatory design, participatory research, codesign, and user-centered design [43,62,63,74,75]. However, in the mental health context, this process often amounts to professionals asking youth about the products they have already designed, with little time or money allocated to the suggested changes that emerge through the process [74,76,77].

We argue that, in the mental health fields, the greatest barrier to adopting a participatory approach, is the implicit paternalistic mindset that may have become ingrained in many academics and practitioners. Mental health researchers and clinicians often assume that young people, especially those who are emotionally vulnerable, do not know when they are suffering and are incapable of asking for the kind of help they need [7,78,79]. However, most youth with anxious and depressive symptoms are well aware of their vulnerabilities and struggles [80]. The key barrier to improving outcomes for these youth is not their own ignorance of whether they need help or even the kind of help they need, but their ability to find the resources and services that will support and train them in a way that speaks to their preferences and modes of learning [78,80,81]. By recruiting youth with mental health challenges from the outset of the design process in order to teach us how they interact and seek information online, we have a better chance of designing interventions that they will find initially engaging, will retain their attention, and will ultimately be viewed as relevant to their needs [46].

In our work, we have taken on this empathy-driven, participatory approach to fundamentally change our starting point in applied game design. Participatory design starts very early, even before any programming of intervention games has begun, by using paper prototyping methods, interviews, and focus groups. Traditionally, this user-research phase is rushed through to reach the “real science.” However, we argue that the scientific outcomes we seek to enhance will not be realized unless empathic, participatory methods are placed at the forefront of our process.

A specific example from our laboratory may be useful to clarify the advantages of using an empathy-driven approach for digital intervention design. In this project, we aimed to design and test a game to help young people quit smoking. Although this game did not directly target mental health, the example is illustrative of the principles and practices that are entirely relevant to mental health applications and useful for clarifying the practices we previously described in the Empathy section. This example is especially relevant to mental health, as the design of this applied game capitalizes on the social peer structure of youth. We tried to increase motivation, commitment, and engagement through game-based experiences that were fundamentally interactive and brought them together with like-minded peers. These social processes are equally important in the design of interventions for anxious and depressed youth, as social ties play a beneficial role in maintaining psychological well-being and mental health [82,83].

Before designing this applied game, we invited young people who smoke to talk specifically about their smoking experiences and how they feel about quitting. Past research on smoking cessation claims that because young people have only just started smoking, they are not motivated to quit [84]. Thus, psychoeducational programs that outline the negative consequences of smoking are the most common intervention approach; skill training based on CBT techniques and motivational interviewing are traditionally employed as well [85-87]. However, none of these interventions have been successful [85,87].

We used a different approach by using structured tools from DT with young people who smoke (eg, card-sort tasks, screen-shot photos of youths’ own phones, and interview protocols; d-school resources [88]). The insights we reviewed were all gained from qualitative interviews with young smokers that were part of unpublished user research with early versions of the game. Our aim was to discover previously misunderstood or overlooked factors that could contribute to designing an end-to-end intervention experience that would effectively help youth quit smoking. We learned that contrary to common assumptions, youth are well aware of the negative consequences of smoking and are often motivated to quit [89-91]. Despite their motivation to quit, they did not know where to look for help; there are no evidence-based interventions available thus far for young people attempting to quit [85,87]. They explained their feelings of inferiority and anxiety that accompany failed attempts to quit. Considering the stigma associated with smoking, they resist asking for help with their addictive vulnerabilities, at least from adults. They are aware that they are struggling and some seek help online anonymously. However, the advice they receive online is perceived as didactic, outdated, and boring.

Instead of focusing on the unhealthy and harmful outcomes of smoking, an empathy-driven lens led us to delve deeper into the emotional and social contexts from which smoking behaviors emerge. We attempted to understand what smoking meant to these young people, how it served important needs, and where they felt that smoking blocked their goals. We discovered that there was a great deal of variability in terms of where and when young people chose to smoke, suggesting the importance of tailoring a DMH intervention to these individual preferences. We also learned that smoking served several functions: to cope with stress; to overcome boredom during the day (eg, waiting for the bus); and crucially, to socialize with friends during breaks. These functional and motivational accounts of young smokers served as the essential scaffold on which we based other evidence-based practices, such as inhibition training [92].

**Figure 1:**
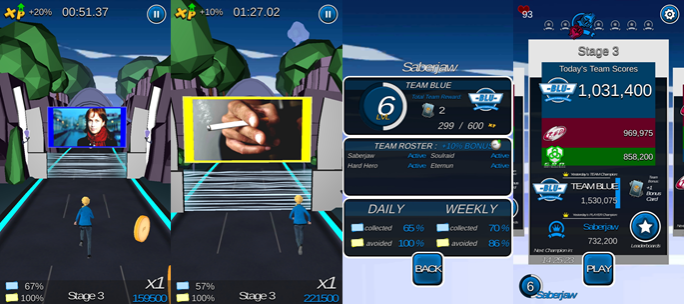
Screenshots of HitnRun showing the runner game and leaderboards.

From these empathy-focused conversations, we designed an intervention to serve as a functional replacement for the smoking habit. We developed the game as a “casual runner,” a genre that lends itself to short bursts of intensely engaging gameplay (ie, 3-5 minutes per session, which is the approximate time taken to overcome a craving moment or smoke a cigarette). To address the problems with the one-size-fits-all approach, we ensured that the game could be played during individualized moments of high craving or boredom. We designed tailored prompts that reminded users to play at instances when they reported experiencing high levels of craving. To enhance relevance in youths’ everyday lives, the game is played on mobile devices, so that young people had access to it whenever they might want to smoke. Figure 1 presents screenshots and a leaderboard example.

We learned how important it was to bring their peer network into the intervention context. We brought them together with like-minded peers who smoked but were motivated to quit through cooperative (and competitive) team-based gameplay that mimicked other online social games with which they were already familiar. Through the cooperative team-based design, youth could learn that there were many like-minded peers that experience the same problems they do, and they could playfully apply “friendly” peer pressure to encourage each other to play the game, which implicitly indicated that they were all quitting together. The competitive elements helped them stay motivated and focused on quitting without resorting to didactic or stigmatizing scare tactics.

RCTs to test the efficacy of this new approach and whether the specific design elements mediate efficacy are underway. We do not have these data yet. However, our main aim of elaborating this example was to provide concrete instantiations of design decisions that would not have otherwise emerged without an empathy-based DT approach.

#### Multidisciplinary Ideation

DT places immense value on cross- and interdisciplinary collaborations with the conviction that true innovation can only arise through a multiplicity of perspectives. A crucial part of DT practices is the generation of a large set of ideas without evaluating the veracity of those ideas in the initial phases and simply collecting the broadest range possible. This approach is in stark contrast to the approach most scientists take, starting from a place of established principles and evidence-based techniques. Although we strongly believe that scientific principles and practices should form the basis of DMH interventions, the potential for new opportunities to engage and retain young people’s attention and time may stem from allowing teams to creatively explore options outside of these empirically established methods. Such exploration is much more likely to yield genuinely novel design possibilities when diverse perspectives are encouraged and then culled via scientific constraints.

We suggest a wide multidisciplinary approach to use DT practices for the development of immersive digital products for young people’s mental health. For example, in our work, we cultivate collaborations among developmental psychologists, neuroscientists, veteran game developers who have extensive experience in the commercial game industry, programmers, and artists, all of whom need to learn each other’s domain-specific language and codevelop a set of shared terms and goals. For the DT methods to work, it is important to invite stakeholders such as teachers, clinicians, physicians, parents, and children themselves to be a part of the codesign process. Through this approach, we can integrate empirically validated principles of clinical change with evocative art and design to render user experiences that are enriching, engaging, and “sticky” enough to bring young people back for more.

The application of the DT framework to DMH interventions requires ideation from more than designers, artists, and mental health professionals. Programmers and formally trained engineers are also crucial partners. Most often, technology and technical requirements are ignored by social scientists. However, early and frequent collaborations with engineers during the early design and evaluation stages are critical, because this is when the back-end, data-acquisition system can be seamlessly integrated with the front-end user interface. This back-end architecture can prove incredibly useful for researchers and clinicians alike. For example, strong, effectively designed data-acquisition systems can be designed to automatically calculate and quantify real-time in-game (or in-app) play or usage behavior. Information about what parts users interacted with, how long they engaged, when they returned, in what areas they lingered longest, how quickly they acquired skills, and other such parameters can serve as powerful analytic tools for the researcher and clinician. Thus, engineers who can build analytic, noninvasive systems can address some of the most pernicious limitations of conventional DMH interventions: participant and client accountability, fidelity, and tracking. In addition, technical experts need to be involved beyond the development and efficacy testing in order to update software continuously (to keep it current and more engaging) and ensure compatibility with changing technology ecosystems (eg, new operating systems and various platforms such as phones, watches, and tablets).

#### Experimentation

“Design thinking is a misnomer; it is more about doing than thinking.” [88]. It seems peculiar to explain experimentation to researchers, but in the context of DT, the meaning of experimentation is different from applying a scientific method in a controlled environment in which one, or very few, factors are manipulated to test a hypothesis. Experimentation in DT refers to a set of processes and practices built around prototyping. A *prototype* is a simplified version of a product, or part of a product, that is created in minimal time and at minimal cost. It is used to test the validity of ideas or design assumptions as rapidly and cheaply as possible. Designers often emphasize the massive advantages of “just doing” (ie, acting out ideas to test their utility before a great amount has been invested in a product or service). In the case of DMH interventions, this prototyping phase is often skipped or applied at such a late stage that only little adjustment is feasible.

Prototyping takes various forms (eg, paper-and-pencil games, whiteboards with sticky notes that depict the flow of a digital experience, storyboards that illustrate the “beats” of a user’s end-to-end experience, and presentation mock ups to click through to get the feel of a tool). All these forms are concrete, tangible artifacts that allow hands-on experience and evaluation before any programming starts and are usually applied iteratively with a small number of target users. Importantly, prototyping is not meant to replace scientifically rigorous experiments or clinical trials; rather, it addresses specific design questions. The results of prototyping iteratively and rapidly are action-based insights about the feel and usability of a product. Often, what emerge are “creative serendipity” and unanticipated insights.

**Figure 2:**
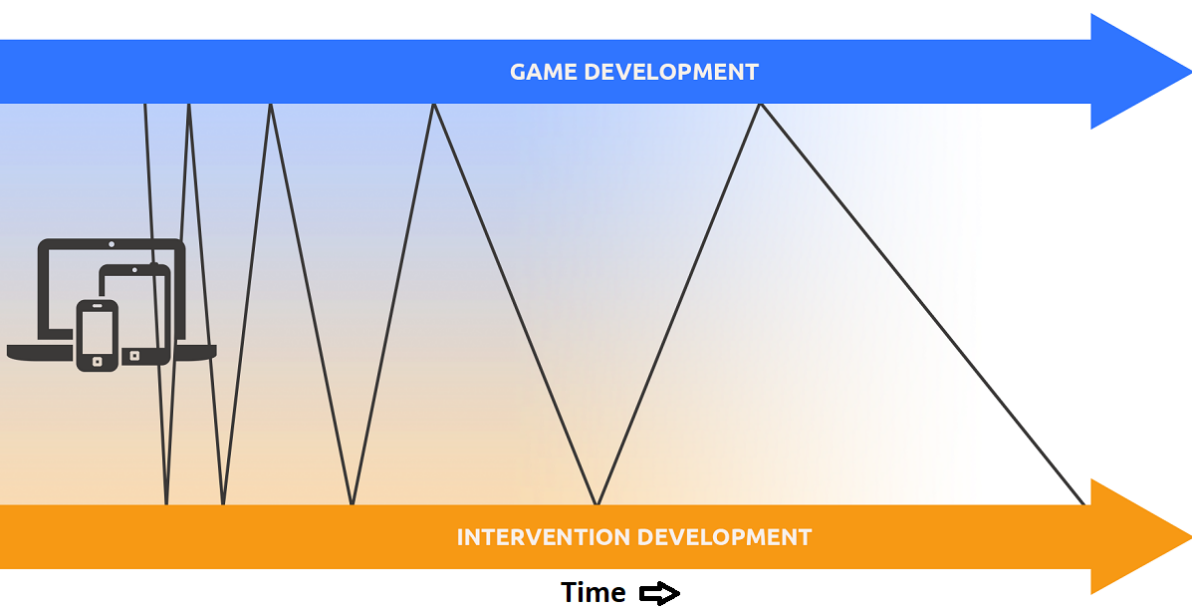
Separate but interactive development timelines for game and intervention goals, with more frequent testing and iterative prototyping at the start of the process than at the end.

One of the most important lessons we have learned is that throughout the experimentation process (prototyping and later phases), it is crucial to separate and synchronize the goals related to the digital tool versus the intervention. We have studied game design, in particular, and we will focus on that domain for articulating our points, but the same principles apply to any interactive app, dynamic website, or other digital media form. The timelines for game development and intervention development run in parallel (Figure 2). Importantly, these streams iteratively influence one another over time. Each domain has its own set of testing principles and practices that are to be applied differentially at each phase.

**Figure 3:**
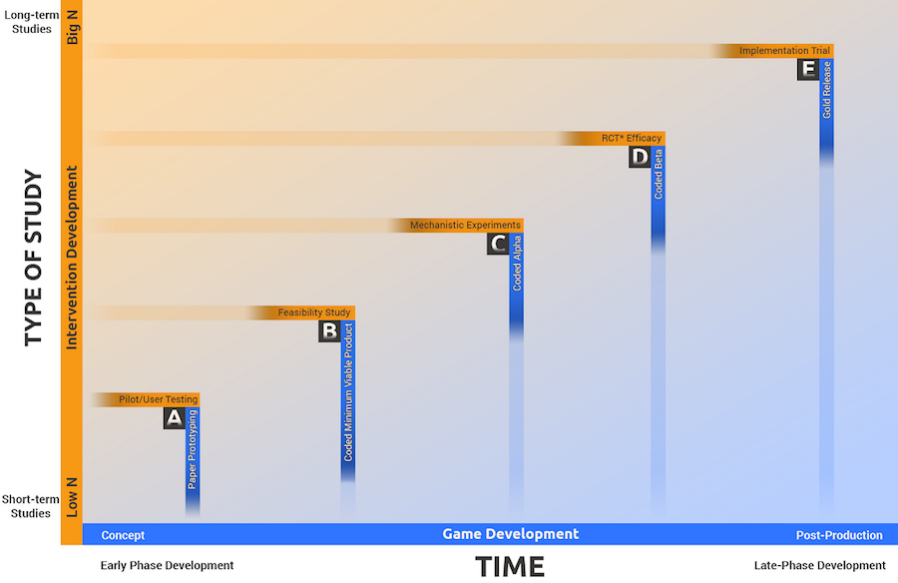
Interaction of the timeline and scope of game development with intervention development. RCT: randomized controlled trial.

For example, early in the prototyping phase, two sets of goals are evaluated in parallel (Figure 3). On the game-development level, we evaluate whether the game’s mechanics (ie, “verbs” of the game) actually work as they were designed (what players actually do to move through the game towards specified goals; this could also be navigation procedures for a website or app). At this early stage, we test whether players proceed through the intended pathways. Do they know what to do to solve a puzzle? Do the controls feel natural? Concomitantly, on the intervention-development level, this phase is often referred to as piloting and can include tests of whether the game elicits the emotional responses intended. Is the cognitive load overwhelming (a barrier with conventional digital interventions for youth)? Do players respond with reactance (ie, backlash or negative affect experienced in response to unsolicited advice)? Do they experience the game as didactic or pedantic and quickly turn it off? Figure 3 also shows the relation between the scope of data collection (eg, sample size), the timing of evaluations, and the different foci and products over the course of the development process. All the prototyping and testing discussed so far fall under Box A in Figure 3. An example from our laboratory with an applied game that has undergone most of the phases in Figure 3 is presented next.

#### Example: MindLight, an Anxiety-Prevention Game for Children

**Figure 4:**
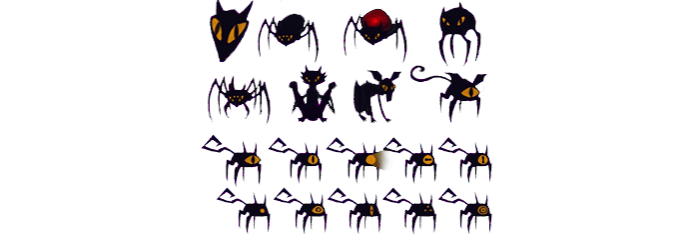
Cat concept design for MindLight.

During the development of MindLight, a game designed to decrease anxiety symptoms in children, a great deal of prototyping was performed to address the two streams of design goals. For example, the game relied largely on exposure techniques to train anxious children to practice facing fears while using relaxation methods. The artists on the project drew several versions of the monsters in the game (Figure 4), given the importance of these figures for triggering fear, and tested whether children would approach them after a certain period of hesitation (game-development goal; Figure 3). The psychologists on the project tested children’s fear responses and appraisals of control to overcome their fear of each of these creatures (intervention-development goal). Contrary to the expectations, most children found the one-eyed monsters humorous and “cute.” Thus, we chose to use a two-eyed creatures instead, to ensure we triggered the fearful responses essential for exposure techniques to work at the intervention level.

Another example of a game mechanic that needed repeated prototyping was neurofeedback. We designed the game so that the calmer children felt while using relaxation techniques during exposure to fearful events (measured by a one-channel electroencephalography system [93,94]), the brighter the light in the game would shine; the more anxious the child felt, the more the light dimmed. A sensitively tuned threshold for when the light would turn on had to be established: players needed to feel motivated when it was dark to practice relaxation skills and maintain motivation to regain their calm, but they could not be so afraid or frustrated that they quit early. Pilot studies helped us identify this threshold as well as a reasonable pace of increasing the threshold over the course of the game while maintaining challenge and engagement.

After several iterations to tweak the dynamic adjustment and reward system, a redesigned beta version (full game coded with 8 hours of gameplay) was used in a series of RCTs. During this phase (D in Figure 3), the main intervention goals were to use rigorous experimental designs to test the game’s impact on children’s anxiety symptoms. Results from four RCTs were reassuring: The data consistently showed significant decreases in children’s anxiety symptoms, with two of the studies showing similar improvements as active control [94] and treatment -as-usual [95] studies and two studies showing improvements equivalent to cognitive-behavioral interventions [93,96], even after long-term follow-up [93].

At the same time, we tested critical elements at the game- development level, including replay ability, engagement, and the likeliness that children would recommend the game to others. Data showed that children were equally likely to recommend MindLight to a friend as one of the most popular commercial games for this age group [94], and they consistently rated the game as fun and engaging [93], suggesting that our prototyping phase was successful. Mediation studies were also performed to examine whether the training mechanics that were designed based on evidence-based techniques (eg, exposure and light-based neurofeedback) were the action mechanisms that explained outcomes and determine if the results confirmed our hypotheses [97]. As expected, children reported feeling fearful of the monsters in the game (ie, exposure worked). More importantly, *increases* in children’s capacity to shine their “mindlight” (the light indicating relaxation measured with neurofeedback) across game sessions predicted reductions in anxiety symptoms 3 months later.

We presented MindLight in this paper as an example to illustrate how the framework in Figure 3 can be concretely applied to the development and research of a digital intervention tool for mental health. We also attempted to highlight how the DT framework was integral to developing an effective digital anxiety intervention tool that was eagerly played by children repeatedly. Importantly, this framework should be applicable to a wide range of digital interventions and is certainly not restricted to applied game development.

## Implementation Considerations

The last stage of Figure 3 is the implementation phase with the “gold release” version of the DMH product, adjusted with insights from the RCTs and mediation studies and polished for distribution purposes. Related to the second DT tenet, rolling out DMH interventions requires a multidisciplinary effort [98]. For digital tools, in particular, we may need to engage more people than stakeholders and policy makers and consider the unique expertise of marketing experts, business leaders, and technical support teams. There are crucial issues to be considered with commercially oriented partners (eg, scientific integrity and conflicts of interest), but if scalability and broad impact are the aims, marketing and business experts may be key to developing optimal models of service delivery. In this final stage, at the game-development level, it is important to consider whether young people discover the games we develop on their own through their own online search initiatives; whether they are interested in engaging with our content; whether we can retain that attention and motivate them to practice skills; and the extent to which they share these DMH programs with peers and family that might benefit similarly from them. On the intervention- development level, implementation tests may need to go beyond RCTs. Current technologies rapidly change in a few years, and there is no reason to believe this rate of change will slow down. In the midst of this rapidly shifting technological landscape, the traditional research designs that require interventions to remain stable across many years may be less practical, useful, and feasible [48,99,100]. Researchers are reconceptualizing the scientific framework, methodology, and implementation strategies that might better suit implementation and outcome studies in the DMH context [48,99,100]. DT and its evaluation practices, with their focus on qualitative and participatory studies, seem to have some useful recommendations in this regard.

## Conclusions

Several reviews have indicated the enormous potential of technology to improve effectiveness, efficiency, cost, reach, personalization, and appeal of mental health interventions for young people. However, significant challenges including engagement, retention, fidelity, lack of personalization, and cognitive load continue to hinder progress in this field. Thus far, all meta-analyses and reviews have highlighted these barriers but have not offered any avenues for actionable solutions [54-56]. We introduced three tenets of DT—empathy, multidisciplinary ideation, and experimentation—and showed how these mindsets and practices can inform the development of future digital interventions. We also provided concrete examples from our work to demonstrate how this new approach can be implemented for young people and provided some preliminary evidence that it can improve outcomes and have an impact on engagement. Ultimately, we argued that integrating DT mindsets and practices with conventional scientific approaches is a promising avenue through which digital tools can address youth mental health. However, we are only at the beginning of merging design and science in the mental health arena. As a discipline, design has been criticized for its lack of quality control, the absence of systems to evaluate the quality, and standardization or documentation of the various DT methods [56,101]. In the future, it will be critical for social scientists and clinical researchers who are interested in appropriating DT to use their scientific standards, methods, and review outlets to evaluate the contribution of DT.

## Abbreviations

CBT: cognitive behavioral therapy
DMH: digital mental health interventions
DT: design thinking
RCT: randomized controlled trial
